# Effects of Electroacupuncture on Syt3 and GluA2 in Rats With Limb Spasms After Intracerebral Hemorrhage

**DOI:** 10.1002/brb3.70366

**Published:** 2025-02-28

**Authors:** Xudong Lu, Huiling Ren, Hequn Chen, Guosheng Shi, Xuanbo Luo, Kai Liu, Qinglin Zhao, Dawei Zhao, Changfa Li, Wei Bu

**Affiliations:** ^1^ Basic Medical College Hebei Medical University Shijiazhuang China; ^2^ Department of Neurosurgery The Third Hospital of Hebei Medical University Shijiazhuang China; ^3^ Department of Neurology The Third Hospital of Hebei Medical University Shijiazhuang China; ^4^ Department of Acupuncture The Third Hospital of Hebei Medical University Shijiazhuang China

**Keywords:** electroacupuncture | intracerebral hemorrhage | spasticity

## Abstract

**Background:**

Clinical studies have confirmed that electroacupuncture (EA) has the potential to improve spasticity after intracerebral hemorrhage (ICH), yet its precise mechanism remains unclear. Synaptotagmin‐3 (SYT‐3), by mediating the internalization of the glutamate AMPA receptor GLUA2, may be related to EA's mechanism. This study aims to explore the mechanism by which EA improves limb spasticity after ICH, providing scientific evidence for its clinical application.

**Methods:**

ICH models were established using stereotaxic injection of autologous tail blood into the right striatum. SD rats were randomly divided into Sham, ICH, ICH + SCRAMBLE, EA, and ICH + TAT‐GLUA2‐3Y groups. Rats in the EA group received 30 min of EA treatment daily after ICH. Muscle tone, neurological deficits, and motor function were assessed. After 3 and 7 days of intervention, the motor cortex was dissected for Western blot analysis of SYT‐3, GLUA2, and P‐GLUA2‐Ser880 expression. Immunoprecipitation was used to detect the interaction between SYT‐3 and GLUA2. Nissl staining and NeuN staining were employed to evaluate brain damage. Fluorescence double‐labeling technique was used to observe the expression of SYT‐3 and GLUA2 in the cell membrane and cytoplasm. Transmission electron microscopy was utilized to examine the microstructure of neurons and synapses.

**Results:**

Compared to the ICH group, rats in the EA group showed reduced muscle tone in the left limbs and significant improvement in neurological deficits and motor function. In the ICH + TAT‐GLUA2‐3Y group, the binding of SYT‐3 and GLUA2 was inhibited, spastic symptoms were alleviated, and membrane expression of GLUA2 increased. In the EA group, SYT‐3 levels were significantly reduced, GLUA2 expression increased in the membrane and cytoplasm, and P‐GLUA2‐Ser880 expression decreased. Rats in the EA group showed increased neuron numbers, normal mitochondrial morphology, and improved synaptic structure in Nissl staining, immunofluorescence, and transmission electron microscopy.

**Conclusion:**

EA effectively improves limb spasticity following ICH by inhibiting the SYT‐3/GLUA2 pathway.

## Graphical abstract

The limb spasm after ICH is associated with the expression of Syt3 and GluA2 and the formation of CP‐AMPARs in the upper motor neurons. Electroacupuncture improves spasticity post‐ICH through downregulating the expression of Syt3 and inhibiting the interaction of Syt3 and GluA2.

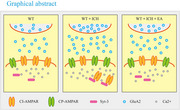

## 1 Introduction

Intracerebral hemorrhage (ICH) is a severe type of stroke characterized by bleeding within the brain parenchyma. Reports indicate that this type of stroke has a global incidence rate ranging from 6.5% to 19.6% (Magid‐Bernstein et al. 2022; Zhu et al. 2019). Recent studies on the mechanisms of ICH have revealed that the brain lymphatic system is involved in the clearance of intracranial hematomas, thereby exerting a positive impact on the recovery of neurological function following hemorrhage (Liao et al. 2024; Chen et al. 2024). Among survivors of ICH, approximately 4% develop spasticity starting from 1–4 weeks after the hemorrhage, and this rate increases to 42.6% within 1–3 months (Wissel et al. 2013). Spasticity is caused by excessive excitation of muscle stretch reflexes, leading to an imbalance in the antagonistic forces of muscles, resulting in abnormal posture and movement control (Chakravarty and Mukherjee 2010). This condition is common after ICH, yet its pathophysiology remains not fully elucidated. Traditional treatments primarily focus on improving spastic paralysis and motor function, including muscle relaxants (Sun et al. 2019), physical therapy (Crozier et al. 2018), noninvasive neuromodulation techniques (Leo et al. 2017), and denervation treatments (Tranchida and Van Heest 2019). However, the efficacy of antispastic drugs is uncertain (Gracies et al. 2015) and often accompanied by side effects such as muscle weakness (Yelnik et al. 2009) or even surgical risks (Kudva et al. 2021). Additionally, factors such as treatment adherence, accessibility of treatment options (Miller et al. 2016), and treatment costs (Mu et al. 2016) are also important considerations. Therefore, finding an alternative, convenient, and cost‐effective treatment method is crucial and urgent. It is necessary to explore new treatment avenues to improve the quality of life for patients with spasticity without altering existing treatment goals.

AMPA receptors are composed of four subunits, GluA1‐4, which can form homomeric or heteromeric tetramers, playing a role in neural signal transmission. Unlike the other three receptor subunits, GluA2 regulates the permeability of AMPA receptors to Ca2+ through modifications at the Q/R site, contributing to the maintenance of intracellular Ca2+ homeostasis (Schröder and da Silva 2023). Synaptotagmin‐3 (SYT‐3) is a calcium‐binding protein belonging to the synaptic marker protein family, primarily localized on the neuronal synaptic vesicle membrane (Weingarten et al. 2022). Recent studies have further identified SYT‐3 in the postsynaptic membrane (Dean et al. 2012; Lu et al. 2023; Awasthi et al. 2019), where it facilitates the endocytosis of the AMPA receptor GLUA2, thereby regulating calcium overload and mitigating glutamate toxicity resulting from the depletion of GLUA2. Therefore, the downregulation of SYT‐3 expression may alleviate intracellular calcium overload and reduce neuronal damage, potentially serving as one of the mechanisms underlying the relief of spasticity following ICH.

Electroacupuncture (EA) therapy integrates traditional acupuncture principles with modern electrotherapy techniques (Liu et al. 2016), showing potential clinical value in treating chronic diseases and pain management in recent years. EA applies a mild electrical current to acupuncture points, combining the therapeutic effects of traditional acupuncture with mechanisms such as regulating the nervous system, promoting blood circulation, and relieving pain, thus significantly enhancing therapeutic outcomes. Studies have shown that EA therapy has potential benefits for patients with neurological diseases, particularly in poststroke rehabilitation (Cai et al. 2017). EA combined with routine care has been demonstrated to provide moderate evidence for treating spasticity following ICH (Chakravarty and Mukherjee 2010). Although these studies indicate that EA has value in treating spasticity after ICH, its precise mechanism of action remains unclear. Therefore, this article aims to investigate the molecular mechanism by which EA improves spasticity after ICH, providing scientific evidence for EA therapy.

## 2 Methods

### 2.1 Animals

This study used healthy male SPF‐grade SD rats weighing 250–280 g, purchased from Beijing Vital River Laboratory Animal Technology Co. Ltd. (Animal License Number: SYXK (Jing): 2021‐0006). The experimental protocol was approved by the Animal Ethics Committee of the Third Hospital of Hebei Medical University (Ethics Number: Z2022‐020‐1).

### 2.2 Establishment of the ICH Rat Model

ICH rat models were created using stereotactic techniques (Cordeiro et al. 2020). Rats were anesthetized with intraperitoneal injection of 1% sodium pentobarbital (40 mg/kg). They were positioned in a stereotactic apparatus, adjusted so that the occlusal plane was 2.4 mm below the interaural plane, and ear bars were fixed to prevent head movement, ensuring that the bregma and lambda were on the same level. The scalp was shaved, and the surgical area was disinfected with 0.5% iodine tincture. A 2 mm midline incision was made between the interaural and interocular lines. After incision, the subcutaneous tissue was bluntly separated to expose the bregma and lambdoid suture. A small hole approximately 2 mm in diameter was drilled 3.3 mm posterior to the bregma and 3.1 mm lateral to the sagittal suture, perpendicular to the skull surface, ensuring no damage to the dura mater or brain tissue. The rat's tail was washed with 40°C water, and after congestion, it was disinfected with ethanol. The tail artery was exposed, and 50 µL of non‐anticoagulated arterial blood was collected using a microsyringe and fixed in the stereotactic apparatus. The syringe was inserted through the hole 8 mm into the right basal ganglia region. Two autologous arterial blood injection methods were used: Initially, 10 µL of arterial blood was injected, the needle was stopped for 2 min, then 40 µL of arterial blood was injected slowly and evenly, the needle was stopped for 4 min, retracted 2 mm, and stopped for an additional 4 min before slowly withdrawing completely. During the procedure, the rat's tail was wrapped with sterile gauze for hemostasis, the incision was sutured, and bleeding was controlled with sterile cotton balls. After the procedure, the rat was carefully removed from the stereotactic apparatus, and the scalp was sutured with attention to sterilization.

### 2.3 EA Intervention

Rats in the EA group began receiving EA intervention on the second day after ICH modeling. Acupuncture points were selected as follows: Quuchi (Large Intestine 11 [LI11]) was located at the depression on the lateral side of the elbow joint near the proximal end of the radius, with a needle depth of 4 mm; Zusanli (Stomach 36 [ST36]) was located 5 mm below the fibular head on the outer side of the hind limb knee joint, with a needle depth of 7 mm. EA parameters were set to sparse‐dense waves, with a frequency of 2 Hz/100 Hz, and the current intensity was gradually adjusted until rhythmic contraction and trembling at the acupuncture site occurred without causing vocalization or struggling, with the current intensity approximately 1 mA. Each intervention lasted 30 min and was performed once daily.

### 2.4 TAT‐GLUA2‐3Y Inhibitor Administration

Rats in the ICH + TAT‐GLUA2‐3Y and ICH + SCRAMBLE groups were intraperitoneally injected with TAT‐GLUA2‐3Y (YGRKKRRQRRRYKEGYNVYG, 5 mg/kg) or SCRAMBLE (YGRKKRRQRRRVYKYGGYNE, 5 mg/kg) 1 h before behavior test at 3 or 7 days after ICH.

### 2.5 Open Field Test

The test was conducted in a quiet, dimly lit room between 9:00 a.m. and 5:00 p.m. Rats were allowed to acclimate to the testing environment for 30 min prior to the experiment to minimize interference with their natural behavior. During the test, rats were gently placed in the center of an open field arena and allowed to explore freely for 900 s. A camera positioned above the arena recorded the rats' movement distance (mm), speed (mm/s), and stationary time (s) over 15 min, and video recordings were synchronized. The camera tracked and mapped the rats' movements, creating activity trajectory maps for each rat. After each test, feces were removed, and the arena floor and walls were cleaned with disinfectant, followed by 75% alcohol, and allowed to air dry before placing the next rat.

### 2.6 Modified Neurological Severity Score (mNSS) Score

Motor, balance, and reflex functions were assessed using the mNSS at 3 and 7 days after ICH (Xia et al. 2020). Higher scores indicate more severe damage, with 10–14 points representing severe neurological impairment, 6–9 points indicating moderate deficits, and 1–5 points indicating mild impairment.

### 2.7 Modified Ashworth Score

Muscle tone was evaluated using the Modified Ashworth Scale (MAS) at 3 and 7 days after ICH (Bohannon and Smith 1987). Scores ranged from 0 to 4, with higher scores indicating greater muscle tone.

### 2.8 Western Blot

Cortex tissue was extracted from −80°C storage, homogenized with lysis buffer to extract total protein, and protein concentration was measured using the BCA method. Protein samples were prepared, denatured by boiling, and 20 µg of protein per well was loaded for SDS–PAGE electrophoresis. Proteins were transferred to PVDF membranes under the following conditions: current 120 mA, voltage approximately 60 V, and transfer time 60 min. Membranes were blocked for 1 h and incubated overnight at 4°C with primary antibodies against SYT‐3 (1:10,000), GLUA2 (1:1000), P‐GluA2‐Ser880 (1:1000), and actin (1:10,000). After incubating with goat anti‐rabbit secondary antibodies (BE0101, Shenzhen BioEAsy) at room temperature for 1 h, membranes were washed. ImageJ software was used to analyze the grayscale values of target bands for GLUA2, P‐GLUA2, and the internal control actin, and statistical analysis was performed on intergroup differences.

### 2.9 Immunofluorescence

Immunostaining of tissue sections used the following primary antibodies: anti‐Neun (1:500), anti‐SYT‐3 (NBP1‐19320, 1:500), and anti‐GLUA2 (Bioss, bs‐1798R, 1:1000). Primary antibodies were incubated overnight at 4°C. Sections were then washed three times with TBS buffer and incubated with FITC‐labeled secondary antibodies for 1 h. Nuclei were stained with DAPI. Sections were observed under a confocal microscope (FV1000, Olympus, Japan), and ImageJ software was used for quantitative analysis.

### 2.10 Immunoprecipitation

Cortex tissue was collected on the third and seventh days post‐ICH to extract total protein. For immunoprecipitation experiments, 1.0 µg of IgG and 20 µL of protein A/G‐agarose beads (Santa Cruz, SC‐2003) were added to the tissue lysate supernatant and incubated at 4°C for 1 h. After centrifugation to remove the precipitate, 5 µL of SYT‐3 antibody (IgG) was added to the supernatant and incubated overnight at 4°C. Immune complexes were affinity‐precipitated using protein A/G‐agarose beads, incubated at 4°C for 2 h, and then centrifuged. The supernatant was discarded, and the pellet was washed four times with 1 mL of cold IP lysis buffer (without inhibitors). After the final wash, the supernatant was carefully discarded, and 40 µL of 1× SDS sample buffer with mercaptoethanol was added, followed by boiling for 10 min. The sample was then centrifuged at 1000 *g* for 5 min at 4°C, and the supernatant was collected. GLUA2 levels were detected using rabbit anti‐GLUA2 polyclonal antibody (Bioss, bs‐1798R, 1:1000) via Western blotting.

### 2.11 Transmission Electron Microscopy

Perfused cortex tissue was cut into approximately 1 mm × 1 mm × 3 mm blocks and fixed in 4% paraformaldehyde solution for 2–4 h at 4°C for prefixation. The tissue was washed in 1/15 M phosphate buffer for 10–15 min, repeated three times. Then, the tissue was fixed in 1% osmium tetroxide for 1–2 h and stored at 4°C. Washing was repeated with 1/15 M phosphate buffer for 10–15 min, three times. Tissue dehydration was performed using graded acetone solutions: 50%, 70%, 80%, and 90% acetone for 10–15 min each, followed by two washes in 100% acetone for 10–15 min each. Embedding was done by mixing embedding medium with 100% acetone in a 1:1 ratio at 37°C for 60 min, then transferring to a 1:3 solution of embedding medium and 100% acetone overnight, and finally embedding in pure embedding medium at 37°C for 5 h. The samples were embedded in capsules or embedding plates and polymerized in an oven at 37°C for 24 h and 60°C for 48 h. Ultrathin sections of approximately 50 nm were cut using an ultramicrotome and stained with uranyl acetate and lead citrate. Synaptic and neuronal structures were observed under a transmission electron microscope.

### 2.12 Statistical Analysis

Data were analyzed using SPSS 20.0 software. Gray values of protein bands and fluorescence intensities were analyzed using ImageJ software. Experimental images were processed and arranged using Photoshop CC2019, and statistical graphs were generated with GraphPad Prism 8.0. Normality of data was tested using the S–W test. Data with normal distribution were expressed as mean ± standard deviation (*x* ± *s*). Group comparisons were performed using one‐way ANOVA; Tukey and LSD methods were used for pairwise comparisons when variance was homogeneous; if variance was not homogeneous, nonparametric tests were applied. A *p* value of <0.05 was considered statistically significant.

## 3 Results

### 3.1 EA Treatment Significantly Alleviates Spasticity

**FIGURE 1:**
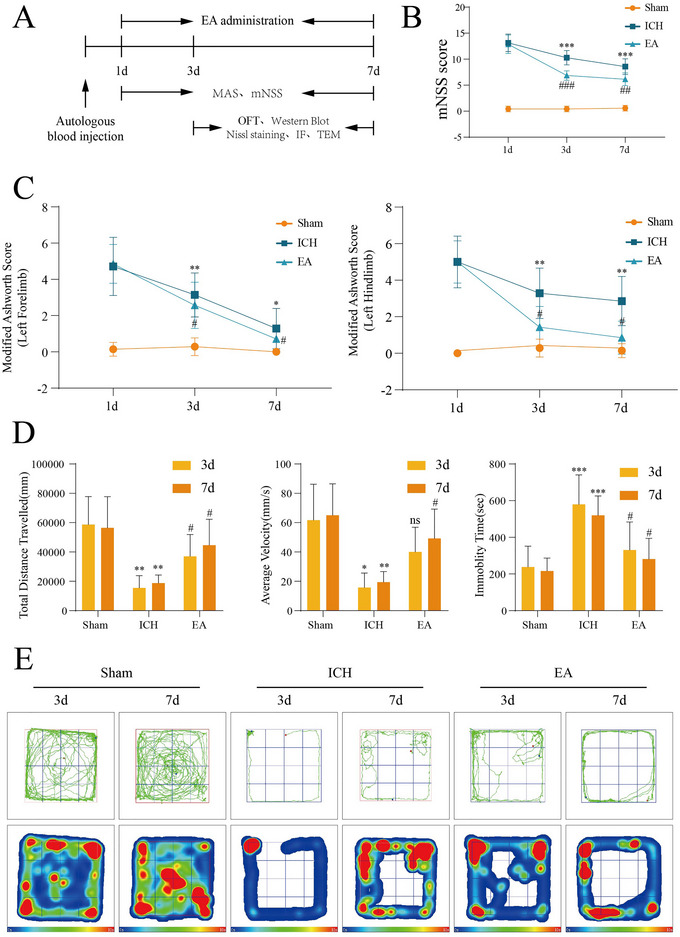
Electroacupuncture improves neurological function, spasticity scores of the contralateral limbs, and motor status following intracerebral hemorrhage (ICH). Experimental design (A) on Days 1, 3, and 7 post‐ICH, muscle tone and neurological function were assessed, and an open field test was conducted on Days 3 and 7. Electroacupuncture treatment commenced on Day 1 post‐modeling, with motor cortex sampling performed on Days 3 and 7. (B) Assessment of neurological deficits (*n* = 7/group). (C) Evaluation of muscle tone in the contralateral upper and lower limbs following ICH (*n* = 7/group). (D) Measurement of motor function (*n* = 7/group). (E) Trajectory and heat maps from the open field experiment. *p < 0.05; **p < 0.01; ***p < 0.001 (Sham vs. ICH groups). #*p* < 0.05; ##*p* < 0.01; ###*p* < 0.001 (ICH vs. EA groups). EA, electroacupuncture.

On Days 1, 3, and 7 after ICH, muscle tone and neurological function were assessed, and motor performance was tested on Days 3 and 7 (Figure 1A). Compared to the Sham group, rats in the ICH group exhibited significant neurological deficits and increased contralateral muscle tone on Days 3 and 7. In contrast, EA treatment markedly improved the neurological deficits (Figure 1B) and increased contralateral muscle tone (Figure 1C) after ICH. ICH group rats displayed a significant reduction in distance traveled, decreased speed, and increased resting time on Days 3 and 7 in open field test (Figure 1D). Conversely, EA group rats showed increased speed and distance traveled and reduced resting time, reflecting improved motor ability. Overall, EA effectively alleviates limb spasticity post‐ICH and enhances motor function.

### 3.2 EA Improves Cortical Neuronal Injury

**FIGURE 2:**
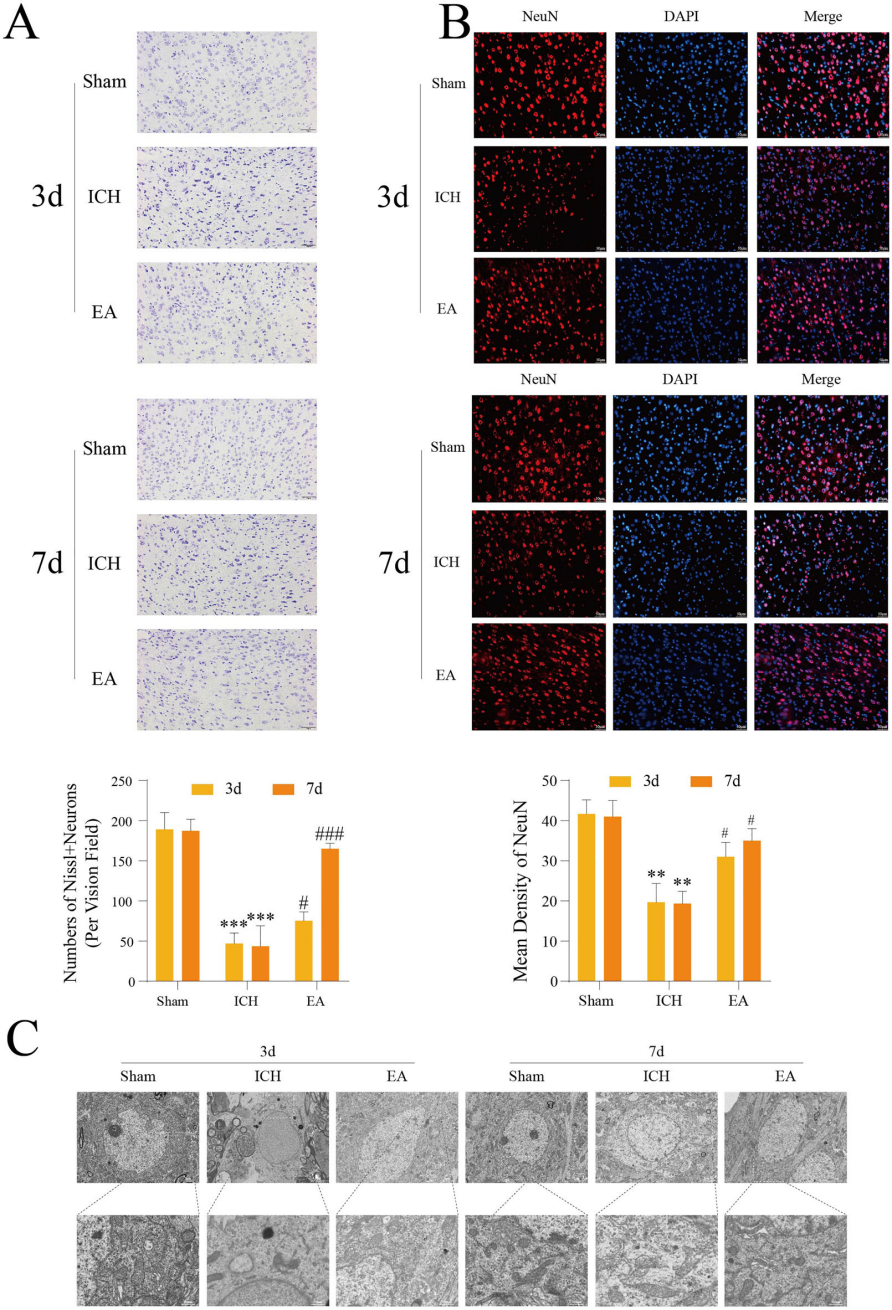
Electroacupuncture (EA) enhances cytoplasmic content, increases the number of healthy neurons, and restores mitochondrial morphology to improve upper motor neurons (UMNs) following intracerebral hemorrhage (ICH). Nissl staining analysis of the motor cortex at 3 and 7 days post‐ICH reveals significant changes (A), with a scale bar of 50 µm (*n* = 4 per group). Additionally, motor cortical NeuN + DAPI staining demonstrates neuronal integrity (B), also with a scale bar of 50 µm (*n* = 4 per group). Mitochondrial morphology in the respective groups was assessed using electron microscopy, as shown in the observation of motor cortical neurons (C) with a scale bar of 2 µm (*n* = 4 per group). Statistical significance is noted as **p < 0.01; ***p < 0.001 (Sham vs. ICH groups), and #*p* < 0.05; ###*p* < 0.001 (ICH vs. EA groups). EA, electroacupuncture.

Furthermore, we explored the effect of EA on upper motor neurons (UMNs). Nissl staining of cortical motor neurons post‐ICH revealed significant cell body shrinkage and reduced cytoplasm (Figure 2A), indicating neuronal damage and functional decline, with extensive pathological changes in motor neurons after ICH. EA intervention significantly reduced neuronal atrophy and restored cytoplasmic staining intensity, with statistically significant differences compared to the ICH group. NeuN immunofluorescence staining (Figure 2B) showed a significant decrease in neuronal fluorescence intensity, indicating a notable decline in neuron number and health. Following EA intervention, NeuN fluorescence staining shows improvement with increased fluorescence intensity in damaged areas and a higher number of labeled neuronal cell bodies. Transmission electron microscopy (Figure 2C) revealed swollen mitochondria in ICH neurons, whereas EA treatment restored mitochondrial morphology to normal. These results indicate that EA improves UMNs damage after ICH by enhancing cytoplasmic content, increasing healthy neuron numbers, and restoring mitochondrial morphology.

### 3.3 Blocking SYT‐3 Molecule Relieves Spasticity

**FIGURE 3:**
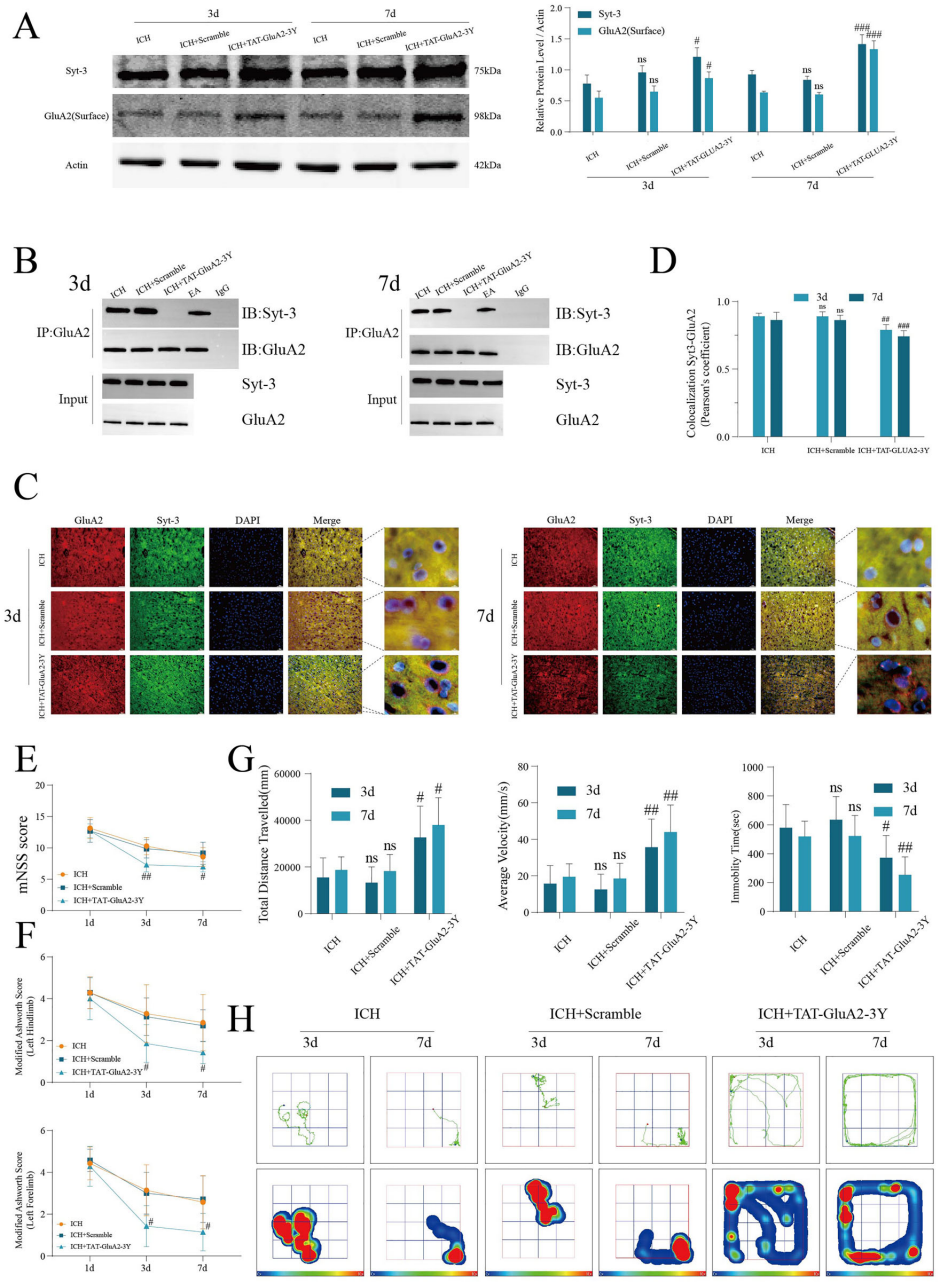
Successful blockade of Syt‐3 improves spasticity in the contralateral limbs of rats following intracerebral hemorrhage (ICH). (A) Western blot analysis demonstrates the expression levels of SYT‐3 and GLUA2 (surface) in cortical neurons at 3 and 7 days post‐modeling (*n* = 6 per group). (B) The interaction between SYT‐3 and GLUA2 was confirmed via co‐immunoprecipitation (*n* = 3 per group). (C) Fluorescent double staining results for SYT‐3 and GLUA2 are presented, with a scale bar of 50 µm, whereas panel (D) illustrates co‐localization results, including the Pearson coefficient (*n* = 4 per group). (E) The modified neurological severity score (MNSS) was utilized for neurological function assessment at 1, 3, and 7 days (*n* = 7 per group). (F) The Modified Ashworth Scale (MAS) was employed to evaluate muscle tone in the contralateral upper and lower limbs following ICH (*n* = 7 per group). (G) Motor function assessments were also conducted (*n* = 7 per group). (H) Trajectory and heat maps from the open field test are included. Statistical significance was determined, with #*p* < 0.05; ##*p* < 0.01; ###*p* < 0.001 (ICH + SCRAMBLE vs. ICH + TAT‐GLUA2‐3Y groups), whereas no statistical significance was observed between the ICH and ICH + SCRAMBLE groups, denoted as “ns.” SYT‐3, Synaptotagmin‐3.

Western blot showed that SYT‐3 expression was higher in ICH + TAT‐GLUA2‐3Y group compared to ICH + SCRAMBLE group, with a significant increase in membrane GLUA2 expression, indicating inhibition of SYT‐3 function (Figure 3A). Immunoprecipitation experiments (Figure 3B) revealed a reduction in the interaction between SYT‐3 and GLUA2 in the ICH+TAT‐GLUA2‐3Y group exhibiting reduced SYT‐3‐GLUA2 binding and suggesting that TAT‐GLUA2‐3Y affects GLUA2 regulation by inhibiting SYT‐3 function. Fluorescence double‐labeling of SYT‐3 and GLUA2 (Figure 3C) further confirmed this result, with significantly reduced co‐localization fluorescence signal areas in the cortex neurons of the ICH + TAT‐GLUA2‐3Y group compared to the ICH and ICH + ICH + SCRAMBLE groups (Figure 3D), with no differences observed between the ICH and ICH + ICH + SCRAMBLE groups. These results indicate that ICH + TAT‐GLUA2‐3Y effectively blocks the interaction between SYT‐3 and GLUA2. Subsequently, motor ability and limb spasticity were assessed in each group. mNSS scores (Figure 3E) and Modified Ashworth scores (Figure 3F) indicated that, on Days 3 and 7 after ICH, ICH + TAT‐GLUA2‐3Y group had lower spasticity scores for contralateral forelimbs and hindlimbs, as well as reduced neurological deficits compared to the ICH and ICH + SCRAMBLE groups, with no significant differences on day 1 after ICH. Open field test (Figure 3G) demonstrated that ICH + TAT‐GLUA2‐3Y group exhibited significantly reduced resting time, increased distance traveled, and improved speed on Days 3 and 7, compared to the ICH and ICH + SCRAMBLE groups. Overall, the application of TAT‐GLUA2‐3Y peptide successfully inhibited SYT‐3 function and improved motor function after ICH, while reducing limb spasticity.

### 3.4 EA Suppresses SYT‐3/GLUA2 Pathway to Treat Spasticity

**FIGURE 4:**
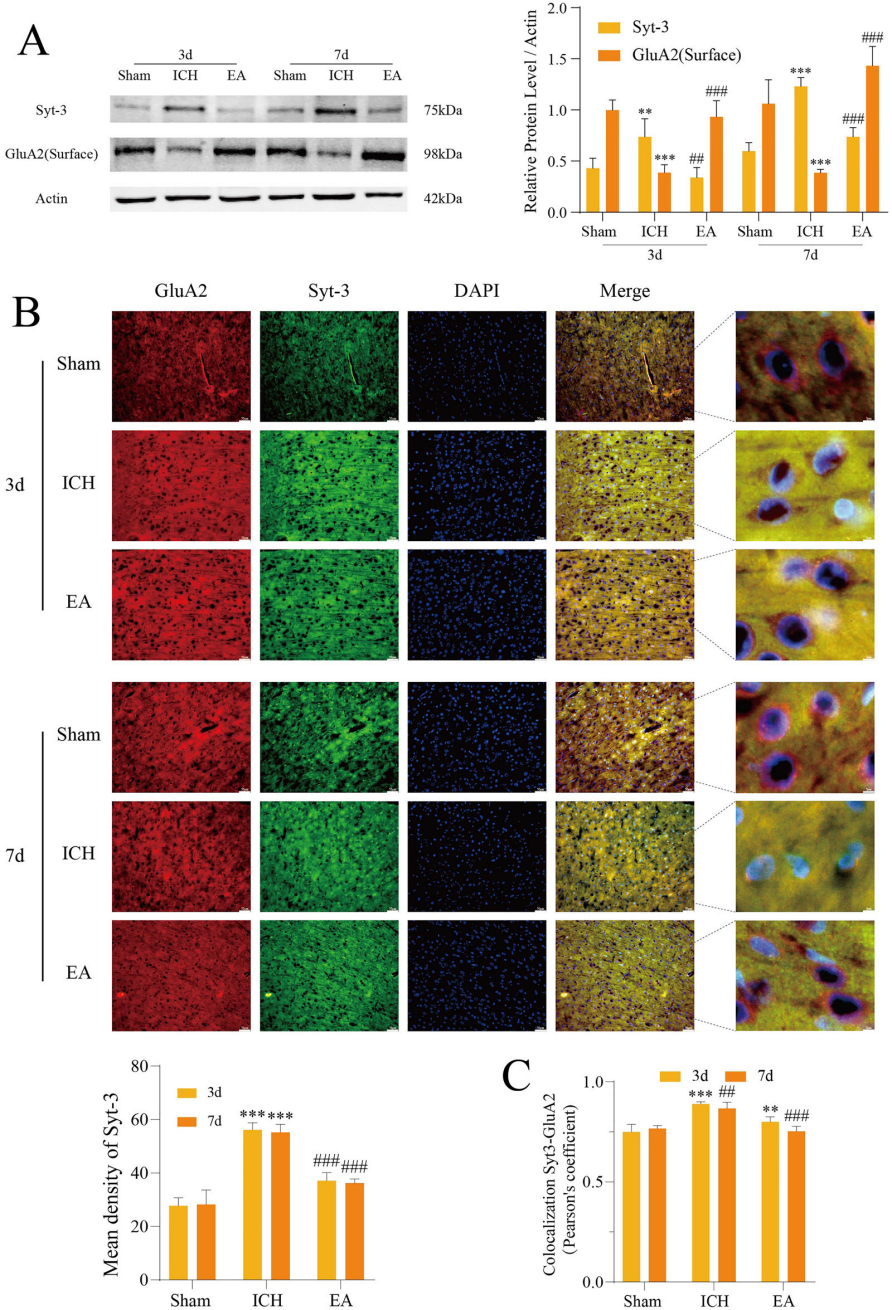
Electroacupuncture inhibits the SYT‐3/GLUA2 pathway by downregulating Syt‐3 expression. (A) Protein levels of SYT‐3 and GLUA2 (surface) were analyzed using Western blotting in upper motor neurons (UMNs) at 3 and 7 days post‐intracerebral hemorrhage (ICH) (*n* = 5 per group). (B) Fluorescent double staining results for SYT‐3 and GLUA2 in cortical motor neurons at 3 and 7 days post‐ICH are shown, with a scale bar of 50 µm (*n* = 4 per group). Panel (C) illustrates the results of co‐localization analysis, including the Pearson coefficient. Statistical significance is indicated as **p < 0.01; ***p < 0.001 (Sham vs. ICH groups) and ##*p* < 0.01; ###*p* < 0.001 (ICH vs. EA groups). EA, electroacupuncture; SYT‐3, synaptotagmin‐3.

Western blot (Figure 4A) confirmed that membrane GLUA2 expression was significantly reduced in the ICH group and SYT‐3 expression was increased. Immunofluorescence analysis (Figure 4B) showed an increase in SYT‐3 fluorescence intensity in the ICH group. Additionally, the EA group exhibited a reduction in SYT‐3‐GLUA2 co‐localization (Figure 4C), which supports the mechanism of SYT‐3 inhibition of GLUA2. Both fluorescence analysis and Western blotting showed that EA treatment led to significantly reduced SYT‐3 and increased GLUA2 levels on Days 3 and 7, suggesting that EA may improve limb spasticity after ICH by suppressing the SYT‐3/GLUA2 pathway. In summary, EA effectively promotes membrane GLUA2 expression by inhibiting SYT‐3 and reducing its internalization effect.

### 3.5 EA Downregulates P‐GLUA2‐Ser880 to Inhibit GLUA2 Endocytosis

**FIGURE 5:**
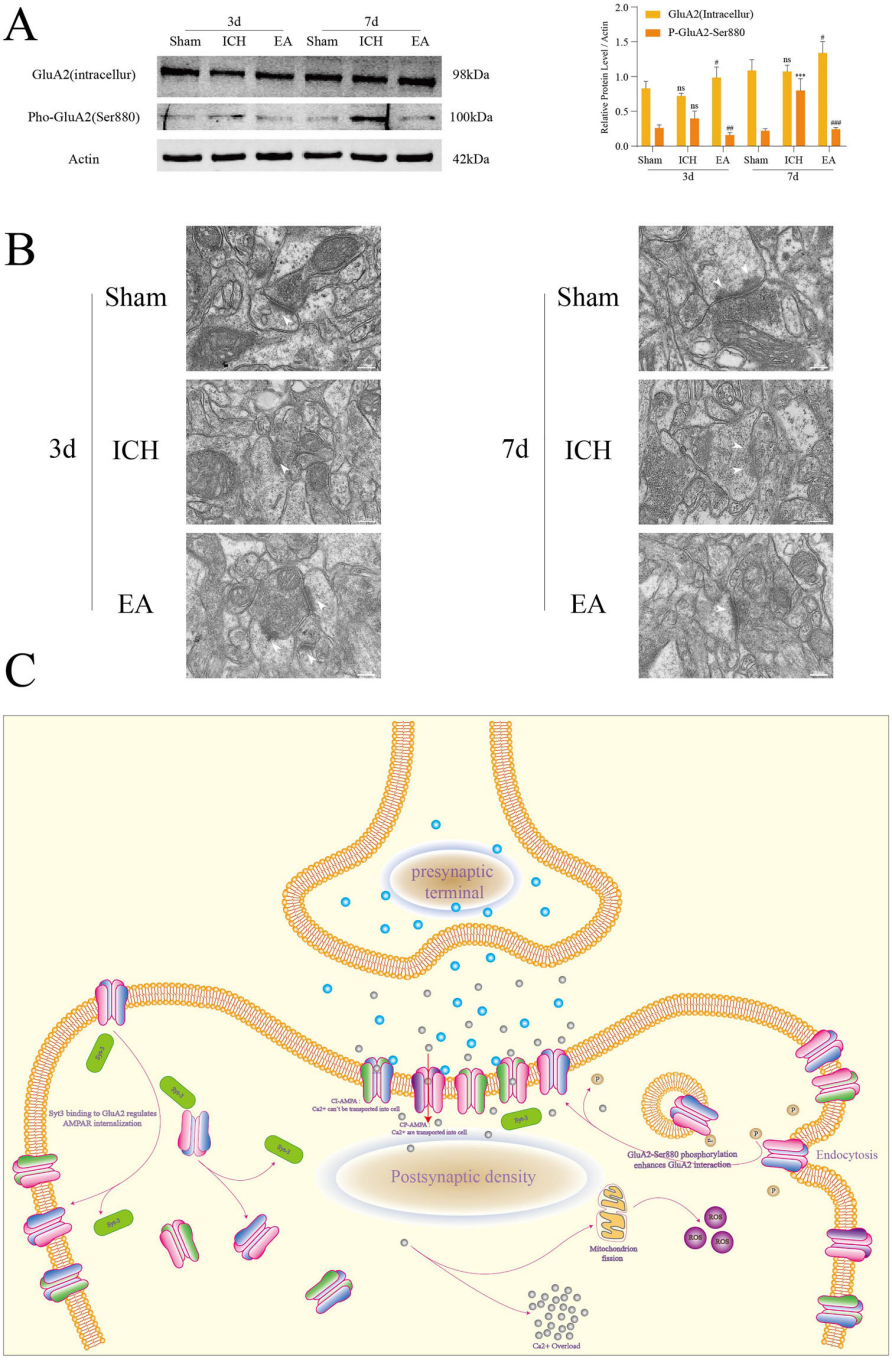
Electroacupuncture (EA) also downregulates P‐GLUA2‐Ser880 and inhibits GLUA2 endocytosis. (A) Western blot analysis reveals the expression levels of GLUA2 (intracellular) and P‐GLUA2‐Ser880 in cortical neurons across groups (*n* = 5 per group). (B) Electron microscopy images depict the postsynaptic density, with a scale bar of 200 nm. (C) This experiment explores the molecular mechanisms involved. Statistical significance is indicated as ***p < 0.001 (Sham vs. ICH groups), with no statistical significance noted as “ns” (Sham vs. ICH groups). Additional comparisons show #*p* < 0.05; ##*p* < 0.01; ###*p* < 0.001 (ICH vs. EA groups). ICH, intracerebral hemorrhage.

To further explore whether GLUA2 endocytosis in spasticity after ICH is entirely mediated by SYT‐3, we investigated the phosphorylation of GLUA2 at Ser880. Western blot (Figure 5A) showed an increase in P‐GLUA2‐Ser880 in the ICH group on Day 7, with no significant difference on Day 3. EA treatment reversed this effect, suggesting that EA may inhibit the formation of P‐GLUA2‐Ser880 and thus prevent GLUA2 translocation from the membrane to the cytoplasm. Transmission electron microscopy (Figure 5B) showed reduced postsynaptic density and expanded synaptic clefts in the ICH group, indicating altered synaptic function and potential impact on neural conduction efficiency. Following EA treatment, synaptic structure was markedly improved, with an increase in postsynaptic density and near‐normalization of synaptic clefts. In conclusion, EA effectively improves cortical motor neuron synaptic structure by downregulating P‐GLUA2‐Ser880 formation, inhibiting GLUA2 endocytosis, and restoring normal synaptic morphology and function, thereby alleviating ICH‐induced neurological deficits.

## 4 Discussion

In this study, we elucidated a potential mechanism underlying spasticity following ICH, specifically the endocytosis of GLUA2 mediated by SYT‐3 in cortical motor neurons, which results in intracellular Ca2+ overload and damage to UMNs. Furthermore, EA was found to reduce SYT‐3 expression, thereby inhibiting the pathogenic role of the SYT‐3/GLUA2 pathway in ICH‐induced spasticity. EA treatment was also associated with increased membrane GLUA2 expression, which appears to be mediated by the suppression of P‐GLUA2‐Ser880 phosphorylation, leading to reduced GLUA2 internalization. These findings provide further evidence for the therapeutic efficacy of EA in alleviating spasticity.

Acupuncture, originating from traditional Chinese medicine, is a simple, cost‐effective, and safe therapeutic modality that has been employed for functional recovery following central nervous system injuries in Asian countries for many years. Recently, acupuncture has also garnered increasing attention in Western countries (Yang et al. 2015; Martens et al. 2017; Karpatkin et al. 2014). Research indicates that acupuncture can effectively enhance limb motor function and reduce the severity of spasticity (Lv et al. 2021; Liu et al. 2014). According to the theory of “treating spasticity with the Yangming meridians as the primary focus,” the Hand Yangming Large Intestine Meridian and the Foot Yangming Stomach Meridian are principal channels, with ST36 and LI11 being commonly used acupoints for treating upper and lower limb spasticity (Tao et al. 2016; Li et al. 2020). EA, which combines traditional acupuncture with modern electrical stimulation techniques, enhances the therapeutic effects by applying controllable electrical currents (Liu et al. 2016). Besides, EA combined with routine care has been demonstrated to provide moderate evidence for treating spasticity following ICH (Chakravarty and Mukherjee 2010).Currently, the mechanisms by which EA alleviates spasticity primarily involve its role in modulating the pain‐spasm cycle (Shin et al. 2009; Lee et al. 2003; Rabinstein and Shulman 2003), spinal cord neuron activity (Fink et al. 2004), and inhibiting the release of inflammatory cells (Qi et al. 2014). However, despite existing research on the regulation of spinal cord neurons by UMNs in ICH (Chakravarty and Mukherjee 2010), studies focusing on UMNs themselves remain relatively limited.

Glutamate is the primary excitatory neurotransmitter in the central nervous system, mediating rapid synaptic transmission through ionotropic receptors such as AMPA receptors (Traynelis et al. 2010; Greger and Mayer 2019). AMPA receptors lacking the GLUA2 subunit cannot effectively block calcium ion (Ca2+) influx due to the absence of the Q/R site modification (Orrenius et al. 2003). This results in excessive calcium ion entry into the cell, disrupting intracellular calcium homeostasis and triggering excitotoxicity, which can lead to cellular damage or death (Schröder and da Silva 2023; Guo and Ma 2021). Excitotoxicity is associated with stroke, traumatic brain injury, and neurodegenerative diseases (Doi et al. 2006; Qin et al. 2022; Smith 2004). However, the connection between spasticity following ICH and glutamate excitotoxicity remains unclear. Our study is the first to demonstrate that EA mitigates spasticity by preserving GLUA2 receptors on the cell membrane, reducing excessive calcium permeability, and preventing neuronal death caused by glutamate toxicity, thereby attenuating damage to the motor conduction system. Furthermore, GLUA2 regulates calcium permeability, which in turn partially suppresses excitatory transmission within the motor system. The observed improvement in spasticity in the EA treatment group provides further evidence supporting this mechanism.

SYT‐3, a calcium‐binding protein, has a structure comprising tandem C2 domains that facilitate binding to calcium ions (Ca2+) (Awasthi et al. 2019; Sutton et al. 1995). In neurons, SYT‐3 primarily functions to sense the influx of calcium ions at the presynaptic terminal. The study by Awasthi et al. (2019) demonstrated that SYT‐3 regulates synaptic strength at the postsynaptic membrane and the CP‐AMPA receptors through its calcium sensitivity, attenuating long‐term potentiation. Additionally, the study investigated its effects on learning and memory processes. Lu et al. (2023) revealed that Syt‐3 enhances cognitive and motor function under ischemia‐reperfusion (I/R) conditions, thereby further elucidating its role in the hippocampus. Through gene knockout and overexpression experiments, they confirmed that reduced Syt‐3 expression in the hippocampus facilitates the recovery of motor function. Our findings contribute to the understanding of SYT‐3's effects on cortical motor neurons and elucidate its further influence on spasticity symptoms following ICH. Further experimental results showed that the application of the SYT‐3 inhibitor ICH + TAT‐GLUA2‐3Y successfully reversed spastic symptoms, suggesting that SYT‐3‐mediated GLUA2 endocytosis is a critical mechanism of limb spasticity post‐ICH. Additionally, EA treatment significantly downregulated SYT‐3 expression after ICH and maintained GLUA2 levels on the cell membrane, indicating that EA improves spasticity by modulating the SYT‐3/GLUA2 pathway. In summary, EA may alleviate ICH‐induced spastic symptoms by inhibiting SYT‐3 function, thereby stabilizing GLUA2 on the cell membrane.

Our study revealed that EA significantly increased the expression of GLUA2 within the cells. To investigate the mechanism underlying this phenomenon, we further analyzed the phosphorylation status of GLUA2. Research indicates that GLUA2 phosphorylation occurs at two key sites: Tyr‐876 and Ser‐880 (Dawson et al. 2010). Phosphorylation at Tyr‐876 promotes the membrane localization of GLUA2 (Hayashi and Huganir 2004), whereas phosphorylation at Ser‐880 facilitates the internalization of GLUA2 from the membrane (Hayashi 2022; Chung et al. 2000). Our data show that EA treatment resulted in a significant reduction in P‐GLUA2‐Ser880 expression, suggesting that EA may inhibit the phosphorylation of GLUA2 at the Ser‐880 site, thereby decreasing GLUA2 internalization and maintaining higher levels of GLUA2 on the cell membrane (Figure 5C). Therefore, we excluded the increase in intracellular GluA2 due to P‐GLUA2‐Ser880 dephosphorylation. However, whether the increase of GluA2 intracellular expression is the initiating factor or mediated by a molecule, or whether both effects exist, remains to be further explored.

In summary, we have identified a critical pathogenic mechanism of acute spasticity following ICH, specifically the cortical neuronal injury mediated by the SYT‐3/GLUA2 pathway. This indicates that SYT‐3 may be a potential therapeutic target for spasticity following ICH, providing experimental evidence for further studies on spasticity and motor disorders after ICH. Furthermore, as inflammation is the most prevalent pathogenic mechanism following ICH, the relationship between the SYT‐3/GluA2 pathway and inflammation requires further exploration (Restivo et al. 2022; Tuttolomondo et al. 2016; Della Corte et al. 2016; Tuttolomondo et al. 2008). Importantly, we found that EA, a traditional Chinese medicine, reduces SYT‐3 expression, inhibits the SYT‐3/GLUA2 pathway, and maintains GLUA2 levels on the cell membrane, thereby preventing cell death caused by glutamate toxicity. This finding supports the theoretical potential of EA for clinical applications.

Our study has several limitations. First, we only assessed the effects of EA on spasticity following ICH without incorporating routine care, which limits a comprehensive evaluation. Additionally, EA intervention was applied only on consecutive days following ICH for 3 or 7 days, without providing insights into long‐term treatment effects. This restricts a full understanding of the long‐term impacts on muscle spasticity and motor impairments. Future research should explore whether lower motor neurons in the motor conduction system are affected and investigate whether SYT‐3 expression in blood or cerebrospinal fluid is associated with spasticity after ICH. SYT‐3 could potentially serve as a predictive marker for spasticity or an indicator for evaluating treatment efficacy.

## Author Contributions

**Xudong Lu**: methodology, writing – original draft, writing – review and editing, investigation, validation, data curation, formal analysis, project administration, visualization. **Huiling Ren**: conceptualization, validation, writing – original draft, writing – review and editing. **Hequn Chen**: methodology, writing – review and editing, data curation, formal analysis. **Guosheng Shi**: methodology, formal analysis, investigation. **Xuanbo Luo**: methodology, data curation, formal analysis. **Kai Liu**: software, data curation. **Qinglin Zhao**: software, methodology. **Dawei Zhao**: writing – review and editing, formal analysis. **Changfa Li**: writing – review and editing, formal analysis. **Wei Bu**: project administration, conceptualization, supervision, resources, writing – review and editing, funding acquisition.

### Peer Review

The peer review history for this article is available at https://publons.com/publon/10.1002/brb3.70366.

## Data Availability

The data that support the findings of this study are available from the corresponding author upon reasonable request.
