# Correction to “[Effects of Electroacupuncture on Syt3 and GluA2 in Rats With Limb Spasms After Intracerebral Hemorrhage]”

**DOI:** 10.1002/brb3.71444

**Published:** 2026-04-17

[Lu X, Ren H, Chen H, Shi G, Luo X, Liu K, Zhao Q, Zhao D, Li C, Bu W. Effects of Electroacupuncture on Syt3 and GluA2 in Rats With Limb Spasms After Intracerebral Hemorrhage. Brain Behav. 2025 Mar;15(3):e70366.]

[In the published version of the article, an error was identified in Figure 5B, where the electron microscopy image corresponding to the 3d Sham group was incorrect; the corrected version of Figure 5 is presented below.]

**FIGURE 5:**
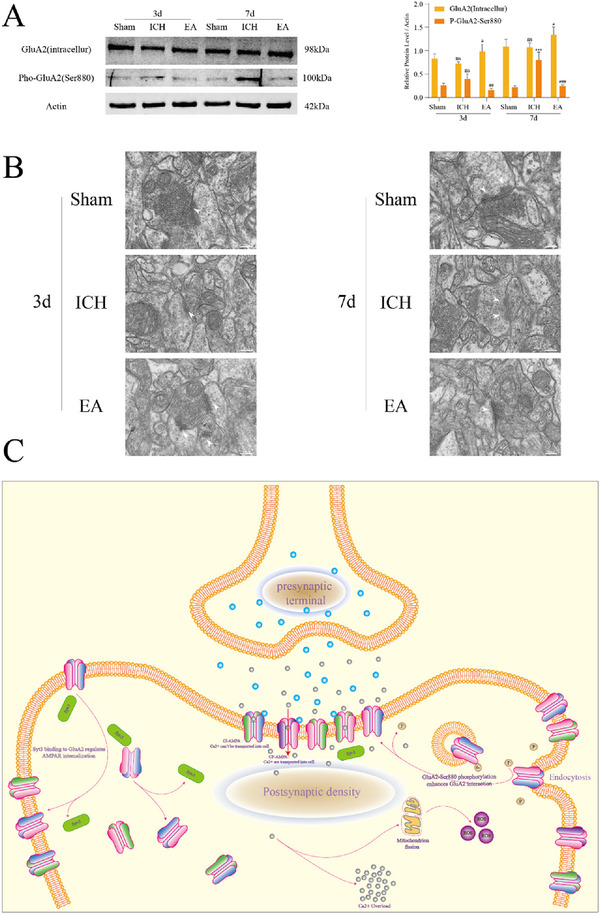
Electroacupuncture (EA) also downregulates P‐GLUA2‐Ser880 and inhibits GLUA2 endocytosis. (A) Western blot analysis reveals the expression levels of GLUA2 (intracellular) and P‐GLUA2‐Ser880 in cortical neurons across groups (*n* = 5 per group). (B) Electron microscopy images depict the postsynaptic density, with a scale bar of 200 nm. (C) This experiment explores the molecular mechanisms involved. Statistical significance is indicated as ***p < 0.001 (Sham vs. ICH groups), with no statistical significance noted as “ns” (Sham vs. ICH groups). Additional comparisons show #p < 0.05; ##p < 0.01; ###p < 0.001 (ICH vs. EA groups). ICH, intracerebral hemorrhage.

We apologize for this error.

